# Mercury Bio-Concentration by Puffballs (*Lycoperdon perlatum*) and Evaluation of Dietary Intake Risks

**DOI:** 10.1007/s00128-012-0788-3

**Published:** 2012-08-18

**Authors:** Jerzy Falandysz, Innocent C. Nnorom, Grażyna Jarzyńska, Dominika Romińska, Kamila Damps

**Affiliations:** 1 Institute of Environmental Sciences and Public Health, University of Gdańsk, 18 Sobieskiego Str, 80–952 Gdańsk, Poland; 2 Environmental Chemistry Unit, Department of Industrial Chemistry, Abia State University, Uturu, Abia State Nigeria

**Keywords:** Bioconcentration, Foods, Forest, Mushrooms, Soils, Wild foods

## Abstract

In the present study, Hg bioconcentration by Puffball (*Lycoperdon perlatum*) mushroom was investigated. Total Hg content of fruiting bodies and topsoil (0–10 cm layer) were determined by cold-vapour atomic absorption spectroscopy. For ten geographically distant sampling sites of Poland, Hg ranged from 0.91 ± 0.28 to 2.4 ± 0.4 (overall range 0.57–4.5) μg/g dry weight in the carpophores and 0.012 ± 0.002 to 0.063 ± 0.024 (overall range 0.0077–0.12) μg/g dw in topsoil. The mean values of the bioconcentration factor ranged from 28 ± 11 to 110 ± 36 (range 9.6–280) indicating that *L*. *perlatum* effectively accumulates Hg and could be classified as a Hg accumulator. Total Hg content of *L*. *perlatum* to some degree seems to be determined both by degree of soil contamination and metal bioavailability to mycelium and also the rate of transfer and accumulation in fruiting bodies at the sites surveyed. Summarized and discussed are published data available on total Hg and methylmercury in *L*. *perlatum*.

Mercury is a global pollutant and remains as one of the most hazardous compounds that contaminate foods and hence impose a high risk to human health (JECFA 2010; USEPA 1987). Mushrooms are a highly biodiverse group of organisms that belong to the kingdom of fungi, and many of them are know as effective accumulators of trace elements (Brzostowski et al. 2011; Falandysz 2008 and 2012; Řanda and Kučera 2004). Mercury is toxic chemical element that is accumulated in mushrooms to great concentration and methylmercury seems even better accumulated that other chemical form of this metal (Alonso et al. 2000; Falandysz et al. 2002a, b; 2003; 2007a, b; 2012a, b; Stijve and Roshhnik 1974).

The puffballs (*Lycoperdales*) comprises of three groups, the *Calvatia* (Giant Puffballs), the *Lycoperdon* (Common Puffballs), and the *Bovista* (Tumbling Puffballs). Puffball *Lycoperdon perlatum* is also known as the Common Puffball, Warted Puffball, Gem-studded Puffball or Devil’s Snuff-box. It is a species of puffball mushroom in the *Agaricaceae* family. The Common Puffballs—the young specimens with white flesh—has long been considered edible and is a culinary favourite in some countries, while the brownish or darker ones are inedible but not poisonous. It is reported to have higher protein content than many other fungi. Mercury contaminations of wild edible mushrooms could pose toxicological risk to consumers because of the toxic nature of Hg species (JECFA 2010).

Total mercury content of mushrooms could be species dependent and varies amongst their ecological groups (saprophytic, symbiotic etc.), and this is what makes data interpretation difficult for different species and areas (Alonso et al. 2000).

The aim of this study was to investigate the contamination with Hg of topsoil and *L. perlatum* collected from ten background and spatially distant sites at the northern part of Poland and to estimate possible Hg intake by consumers. The bioconcentration factor (BCF) values were calculated to estimate the potential of this species to accumulate Hg.

## Materials and Methods

Fruiting bodies (carpophores) of *L. perlatum* and beneath surface layer (0–10 cm) of soils (ca. 100 g) were collected. Sampling of 10 cm-depth horizon was aimed at understand-ing the spatial difference in Hg distribution in the soil substrate. The samples were collected from ten sites in Poland, *i.e*.: Krokowa, Lębork, Lipusz, Kartuzy, Miracho-wo and Łapino at the Pomorskie Voivodeshisp (Pomerania); Kiwity at the Warmia Land (Warmia), Piska forests at the Mazury Land (Masuria), Ciechocinek at Kujawy Land (Kujawsko-Pomorskie Viovodeship) and Poniatowa at the Chodelska Dale (Lubelskie Voivodeship) in 2001–2008 (Table 1).

**Table 1:** Mercury in *L. perlatum* and soils (*μ*g/g dw) and values of the BCF (arithmetic mean, SD, range and median, respectively)

Site and year	Hg		BCF
	Caprophores	Soils	
Krokowa, Pomerania, 2003	2.4 ± 0.4	0.057 ± 0.018	46 ± 14
n = 15 (85)^a^	(1.5–2.8) 2.5	(0.027–0.090) 0.055	(19–70) 44
Lębork, Pomerania, 2007	1.3 ± 0.4	0.043 ± 0.020	36 ± 15
n = 15 (84)	(0.69–2.2) 1.3	(0.025–0.096) 0.039	(9.8–55) 39
Lipusz, Pomerania, 2007	2.0 ± 0.4	0.020 ± 0.007	110 ± 36
n = 15 (47)	(1.2–2.8) 2.0	(0.012–0.040) 0.018	(31–160) 120
Kartuzy, Pomerania, 2007/2008	1.9 ± 0.8	0.047 ± 0.034	100 ± 100
n = 13 (75)	(1.3–3.6) 2.6	(0.010–0.080) 0.049	(25–280) 68
Mirachowo, Pomerania 2007	1.1 ± 0.2	0.046 ± 0.016	28 ± 11
n = 8 (16)	(0.92–1.5) 1.1	(0.031–0.073) 0.037	(13–42) 31
Łapino, Pomerania, 2006/2007	2.0 ± 0.8	0.025 ± 0.007	83 ± 34
n = 13 (105)	(1.3–3.9) 1.7	(0.020–0.045) 0.023	(29–170) 78
Kiwity, Warmia, 2003	2.4 ± 1.1	0.049 ± 0.036	77 ± 47
n = 13 (53)	(1.1–4.5) 2.0	(0.012–0.12) 0.042	(9.6–140) 83
Piska forests, Mazury, 2003	1.3 ± 0.3	0.023 ± 0.005	65 ± 27
n = 15^*b*^	(0.70–1.8) 1.4	(0.013–0.032) 0.022	(22–140) 60
Ciechocinek, Kujawy, 2004	0.91 ± 0.28	0.012 ± 0.002	75 ± 24
n = 15 (58)	(0.57–1.4) 0.82	(0.0077–0.015) 0.013	(47–120) 66
Poniatowa, Chodelska Dale, 2001	1.8 ± 0.5	0.063 ± 0.024	35 ± 23
n = 11	(1.1–2.9) 1.7	(0.029–0.10) 0.066	(12–90) 30

^a^Number of samples and number of fruiting bodies (in parentheses)

^b^Number of fruiting bodies

Fruiting bodies after cleanup from the plant material and particles of soil with a plastic knife were initially air-dried at room temperature in a well-ventilated place for two–three days, and then further oven dried at 65°C for 48–96 h. The specimens were next crushed and ground in an agate mortar to fine powder. Soil substrate samples, after removal of visible organisms, small stones, sticks and leaves were air dried at room temperature for approximately 4–6 weeks and then sieved through a pore size of 2 mm.

Total Hg content of materials was determined by cold-vapor atomic absorption spectroscopy (CV–AAS) after thermal decomposition of sample matrix and further amalgamation and desorption of element from gold wool (Mercury analyzer type MA–2000, Nippon Instruments Corporation, Takatsuki, Japan). Analytical control and quality assurance (AC/QA) were performed through the analysis of certified reference material CS-M-1 (dried fruiting bodies of mushroom Cow Bolete *Suillus bovinus*) produced by the Institute of Nuclear Chemistry and Technology in Warsaw, Poland. Declared content Hg in these certified reference material was 0.174 ± 0.018, while our measurements showed 0.171 ± 0.008 *μ*g/g dw (n = 3). In addition, with every set of 10 mushroom or soil samples examined, one blank sample was included. For mushroom fruiting bodies and soil substrate the limit of detection (LOD) was 0.005 μg Hg/g dw, and the quantitation limit (LOQ) was 0.003 μg Hg/g dw (Jarzyńska and Falandysz 2011).

## Results and Discussion

The results of this study showed varying Hg concentrations in *L. perlatum* ranging from 0.57 to 4.5 *μ*g/g dw with the mean values for the sites investigated ranging from 0.91 ± 0.28 *μ*g/g dw for Ciechocinek to 2.4 ± 0.4 *μ*g/g dw for the sites Krokowa and Kiwity. The range of the median values according to site is 0.82–2.5 *μ*g/g dw for Ciechocinek and Krokowa, respectively (Table 1).

The results of this study are comparable to earlier reports for Hg in *L. perlatum* for specimens from background areas of Sweden, Poland, Slovakia, Switzerland, and Italy (Table 2).

**Table 2:** Mercury in Puffball and soils (*μ*g/g dw) in Europe and values of the BCF (arithmetic mean, SD, range and median, respectively)

Site and year	Hg			References
	Caprophore	Soils	BCF	
Sweden, Umeå, 1995 n-6(30)^a^	1.6 ± 0.7 (0.77–2.8)	0.10 ± 0.13 (0.018–0.25)	65 ± 29 (11–110)	Falandysz et al. (2001)
Poland, Rogóźno, 1984/85 n-1	1.4			Lasota and Witusik (1987)
Poland, Łubiana, Kościerzyna county, 1993 n = 15	1.1 ± 0.4 (0.48–1.9) 1.1			Falandysz et al. (1996)
Poland, Zaborski Landscape Park, 1997/98 n = 16	3.7 ± 1.7 (1.5–6.8)	0.031 ± 0.021 (0.0091–0.072)	160 ± 120 (46–420)	Falandysz et al. (2002a)
	3.3	0.025	120	
Poland, Borecka Forest, 1998 n = 16(146)^a^	3.4 ± 1.3 (1.3–6.1) 3.2	0.020 ± 0.019 (0.009–0.040)	200 ± 91	Falandysz et al. (2002b)
		0.018	190	
Poland, Łukta and Morąg, 1997/98 n = 16(168)^a^	2.8 ± 0.5 (1.3–3.1) 2.2	0.054 ± 0.12 (0.012–0.41) 0.026	89 ± 48 (6.6–210) 78	Falandysz et al. (2003)
Poland, 2001–2007 n = 133(549)^a^	0.91 ± 0.28–2.4 ± 1.1	0.012 ± 0.002–0.063 ± 0.024	28 ± 11–110 ± 36	^b^
	(0.57–4.5); 0.82–2.6	(0.0077–0.12)	(9.6–280)	
Germany n = 2	3.3 (1.5–5.1)			Seeger (1976)
Slovakia	2.0 ± 0.6 (0.3–3.2)			Svoboda et al. (2006)
Bohemia, Precambrian shales	3.5 ± 0.1			Řanda and Kučera (2004)
Bohemia, vicinity of multi-metal smelting plant	4.0 ± 0.1			
Switzerland, Lausanne, contaminated site	16 (6.6–22) 8.6			Quinche (1979)
Switzerland, Lausanne, background site	2.6 (2.3–5.8) 3.8			Quinche (1979)
Switzerland	0.58 [MeHg: 0.012]		6.3 [MeHg: 12]	Reider et al. (2011)
Slovenia n = 2	2.2 (2.1–2.3)			Byrne et al. (1979)
Slovenja, Idrija, Hg melting site n = 1	41			Stegnar et al. (1973)
Slovenja, 5 km from Idrija Hg melting site n = 1	32 [MeHg 3.5]			Stegnar et al. (1973)
Slovenja, Podljubelj n = *6*	27 (20–34) [MeHg: 0.35]	11 (7.1–19)		Stegnar et al. (1973)
Slovenja, Podljubelj n = 7	25 (17–28) [MeHg: 0.35]			Stegnar et al. (1973)
Slovenja, background area n = 3	8.4 (5.3–14) [MeHg: 0.36]	0.55 (0.53–0.58) n = 2		Stegnar et al. (1973)
Italy, Siena n = 1	2.8	0.31	9.0	Bargagli and Baldi (1984)
Italy, Regino di Emilia	1.9 (1.2–2.6)			Cocchi et al. (2006)

^a^Number of samples and number of fruiting bodies (in parentheses)

^b^Present study

Total Hg content of topsoil depending on the site, revealed low contamination with this element and mean concentrations varied between 0.012 ± 0.002 and 0.063 ± 0.024 (overall range 0.0077–0.12) μg/g dry weight in soils (Table 1). The greatest Hg in soil at 0.12 μg/g was observed in a sample from Kiwity, site. None of the surveyed sites was recognized as being under the influence of any point Hg pollution source. These soils Hg data of the present study imply background levels since the range of concentration values reported for forest soils in a boarder study conducted in Poland revealed values usually well below 0.5 *μ*g/g dw (Drewnowska et al. 2012; Falandysz et al. 1996, 2002a, b; 2003; 2007a, b; 2012a, b). Higher soil Hg concentrations of 0.53–0.58, and 7.1–19 μg/g were reported for some soils from Slovenia while 0.31 μg/g in soils from Italy (Table 2).

Based on calculated values of BCF *L. perlatum* could be classified as a good accumulator of Hg as all samples showed very high BCF values (BCF ≥ 10; range 9.6–280) (Table 1). The highest BCF of this study (280), though higher than the maximum BCF values reported by some earlier studies (maximum up to 110 or 210), it is however lower than the maximum BCF of 420 reported for Zaborski Landscape Park (Table 2).

To assess the health risk to consumers due to Hg contained in the *L. perlatum* the mean, median and range concentrations in (Table 1) and reference dose (RfD: 0.0003 mg/kg bw daily) were used in estimating the Provisional Tolerable Weekly Intake (PTWI: 0.004 mg/kg bw) values (JECFA 2010; USEPA 1987). A meal consisting of 300 g of fresh puffball would expose a consumer to Hg dose ranging from 27 μg Hg at the Ciechocinek site to 72 μg Hg at the Krokowa and Kiwity, considering the mean concentration of Hg contents of Puffballs collected from these sites. And these values are 130 % and 340 % of the daily Hg reference doses respectively. The amount of fresh caps needed so as not to exceed this Hg limit for 70 kg body weight individual would be 0.23 kg at Ciechocinek and 0.087 kg at both the Krokowa and the Kiwity sites. Considering the established PTWI value and the mean mushroom Hg contents for the various sites investigated, consumption of a meal made of 300 g of *L. perlatum* for a week could provide depending on the site from 191 μg Hg at Ciechocinek to 504 μg Hg at both the Krokowa and Kiwity, respectively, and this would comprise from 1,900 % to 5,300 % of PTWI. This shows that the consumption of this mushroom could pose health risks to consumers.
